# Pediatric dental treatments with pharmacological and non-pharmacological interventions: a cross‐sectional study

**DOI:** 10.1186/s12903-021-01555-7

**Published:** 2021-04-12

**Authors:** Rania A. Baakdah, Jihan M. Turkistani, Amjad M. Al-Qarni, Asuf N. Al-Abdali, Heba A. Alharbi, Joud A. Bafaqih, Zaina S. Alshehri

**Affiliations:** 1 King Abdulaziz Medical City, National Guard Health Affairs, Jeddah, Saudi Arabia; 2 Alfarabi Dental College, Jeddah, Saudi Arabia; 3 Ibn Sina National College, Jeddah, Saudi Arabia

**Keywords:** Pharmacological, Interventions, Dental treatments, Pediatric patient, Dental care for children, Pediatric dentistry, Child behavior, Conscious sedation, Anesthesia, general

## Abstract

**Objectives:**

Behaviour management strategies involving pharmacological or non-pharmacological interventions during dental procedures should be considered to attain safe and successful treatment outcomes. This study compared the frequencies of use and the completeness of treatment with these interventions.

**Methods:**

A total of 1725 dental records of patients up to 18 years old, who were treated in the King Abdulaziz Medical City in Jeddah City from October 2018 to June 2019, were used in this retrospective, cross-sectional study. Inferential analysis, Chi-square test, Kruskal–Wallis test, and regression model were used in the data analysis.

**Results:**

About two-thirds of the patients were treated with attendant non-pharmacological interventions, while one-third, with pharmacological interventions. The application of General Anesthesia (GA) was the most frequently used intervention. Restorative procedures and extractions were done in higher frequencies with pharmacological interventions. Treatments with space maintainers and orthodontic appliances were carried out in higher frequencies with non-pharmacological strategies. The choice of intervention was significantly influenced by the systemic conditions of the patients. Patients treated with non-pharmacological intervention comprised the dominant type of patients, because they required treatments with less pain. Those treated with GA needed restorative treatments and extractions, or treatments that involve pain, but these treatments had higher frequencies of being completed.

**Conclusions:**

The treatments with pharmacological intervention through GA have higher frequencies of being completed, compared to those with non-pharmacological interventions. Factors, such as age, potential to complete the treatment, and the type of dental treatment applied, influence the choice of treatment intervention.

## Background

The specialization of pediatric dentistry provides both primary and comprehensive oral health needs of infants and children, including those with special health care needs [1]. The provision of dental care is needed in preventing and eliminating orofacial disease, restoring the form and functions of dentition, and correcting facial disfiguration. However, pediatric dentists are confronted by difficult challenges brought about by the patient’s young ages, their behavior during treatment, or their special medical needs [2, 3].

One of the major problems in pediatric dentistry relates to the management of uncooperative and anxious children during treatment. Dental fear and anxiety (DFA), which indicates strong negative emotions associated with dental treatment among children and adolescents, is the most common cause of behavioral management problems and the non-compliance of children during treatment [4]. The prevalence of DFA in children was reported to be 5–20% [5]. Pain is one of the reasons why a patient may be fearful of dental procedures, which is particularly true for pediatric dental patients [6]. Dental pain is caused by an inflammatory condition, tissue damage, infection, or invasive treatment [2]. Therefore, careful pain assessment and attendant control strategies during dental procedure can promote a better relationship between the dentist and the patient by building mutual trust, relieving fear and anxiety of the patient, and enhancing positive attitudes of the patients toward their future visits [7]. Likewise, the children’s medical conditions can cause distinctive challenges in dental treatment, because some medical diseases can influence the timing and the type of dental treatment to be administered, as well as the techniques used in pain and anxiety control [8].

Both pharmacological and non-pharmacological behavioral management are used to perform oral health care safely and efficiently. The American Academy of Pediatric Dentistry (AAPD) had established guidelines on how to conduct a successful dental treatment by reducing pain, fear, and anxiety, to establish a positive attitude toward dental treatment, and to build a trustworthy relationship between the dentist and the patient/parent [2]. The choice of the technique by a skillful practitioner must be customized after fully understanding the cognitive, social, and emotional qualities of the child [2, 8].

The non-pharmacological intervention is divided into two groups: communication and confidence-building and psychotherapeutic strategy. This strategy is based on the concept of learning by changing the unfavorable attitudes of the child in a specific situation, which includes tell-show-do technique, biofeedback, positive reinforcement, hypnosis, distraction, among others [3, 8, 9]. On the other hand, pharmacological interventions include conscious sedation, which is delivered by a variety of means and combinations and general anesthesia (GA) [3, 4]. The tell-show-do technique has shown to be the most frequently used technique, with high parental acceptance, and is the safest method among the non-pharmacological techniques. Sedation by nitrous oxide is the second most favorable method. The hand-over-mouth technique, which is used in some countries, has the lowest acceptance by the parents and by dentists. The AAPD also suggested to minimize the use of this strategy [8, 10, 11].

The use of sedatives through the oral and inhalation routes of administration is a very cost-effective alternative option to general anesthesia. Additionally, it is the most popular technique among dentists and is also preferred by patients [12, 13]. An increase in the use of nitrous oxide reflects the high demands of this sedative substance by the parents, the contemporary education in pediatric dentistry, and the changing quality of the resident’s training [14]. However, a study was done to examine the factors that influenced dentists’ sedation choice when a child presents in pain. It was found that the most important factor in dentist’s decision was dental caries, whereas age and other factors were of lesser importance [15]. Moreover, sedation was an option for patients with multiple morbidities who cannot tolerate dental treatment. Although it is sometimes a challenge for the patients, but with teamwork between the dentist and healthcare provider, a safe and successful dental treatment under sedation can be achieved [16].

Studies that describe and compare the frequencies of each treatment intervention used and the dental procedures that were done through these strategies are rarely published. The findings of a comprehensive systematic review on these strategies revealed the effectiveness of pharmacological and non-pharmacological interventions in managing DFA in children and adolescents during dental procedures. However, further assessments on the efficacy and the safety of using these interventions are needed [4]. It is essential to know about the possible differences in the frequencies of use and preferences of these dental treatment strategies, since children’s age, behavior, and medical conditions can cause distinctive challenges in dental treatment outcomes. Moreover, some medical diseases may affect the timing and the quality of dental care services as well as the strategy used. Addressing this gap is the focus of this study to help professional decisions about the use of pharmacological or non-pharmacological techniques in the dental office. This targeted specific purpose of which dwell on the frequencies of using pharmacological and non-pharmacological interventions as applied to pediatric dental patients of the King Abdulaziz Medical City (KAMC) in Jeddah City and the identification of the factors that may influence the choice, on which intervention and technique are preferred by pediatric dentists.

## Methods

### Study design

This retrospective, cross-sectional study was conducted at the hospital of KAMC in Jeddah, Saudi Arabia. This center is a tertiary care center that serves the employees of the Ministry of National Guard and their eligible dependents. However, eligibility exceptions can be made to non-eligible patients with medically compromised conditions through Prince Noura Oncology Center for oncologic patients and King Fahad Cardiac Center for cardiac patients. This study involved a review of the records of pediatric patients, who attended the dental clinics and operating rooms from October 2018 to June 2019. Pediatric patients were referred from primary care centers, out-patient clinics, and in-patient for dental treatment. Those referred patients were first screened and dental treatment was planned for them using either pharmacological or non-pharmacological techniques taking into consideration their age, medical condition, and cooperation.

### Study sample

The sample size was calculated using the Roasoft sample size calculator [17]. Given a marginal error of 2.5%, a confidence interval of 95%, and a response rate of 50% for those patients who underwent dental treatments using pharmacological intervention, a minimum of 1428 patient records was required to produce statistically accurate results. A total of 1725 pediatric dental records were reviewed for this study, and the sample was selected from them using the non-probability consecutive sampling technique, where every consecutive patient record that met the eligibility criteria was included in the study.

### Eligibility criteria

Pediatric patients of up to 18 years old and patients treated by a pediatric dentist were included in this study. Those pediatric patients with missing records were excluded.

### Data collection

The data extracted from the pediatric patients’ records were entered into a self-designed data collection Excel sheet. This form consists of: (1) demographic information, which include patients’ gender, date of birth and area of residence; (2) medical status of patients whether the patient is medically free or medically compromised; (3) treatment strategies, which is divided into pharmacological and non-pharmacological; (4) treatment procedures either preventive, restorative, surgical or orthodontic procedure each in details; and (5) treatment completion. Treatment completion can be defined as providing the entire planned treatment procedures involving preventive, restorative, orthodontic treatment and extractions, in addition to the number of the visit in order to complete the case.

### Ethical consideration

The ethical approval for the conduct of this study was obtained from the Institutional Review Board of King Abdullah International Medical Research Center.

### Data analysis

Data analyses were done using SAS 9.4, which calculated the frequencies and percentages for the categorical variables (i.e., gender, area of residence, medical condition, treatment strategy type and procedure type, and treatment completion). While, the numerical variables were presented as mean ± SD (i.e., age, number of visits, dental procedure frequency and treatment strategies frequency). Inferential analysis was performed using a combination of parametric and non-parametric methods. The Chi-square test was used to determines whether there is an association between categorical variables such as the association between treatment strategy and gender, medical condition, area of residence, treatment completion, and type of dental treatment provided. The Kruskal–Wallis test, and the logistic regression model were used to determine the association between numerical and categorical variables such as the association between treatment strategy and age, number of treatment visit, and the number of dental treatment services provided. The significance level was set at 0.05% for all statistical tests.

## Results

**Fig. 1:**
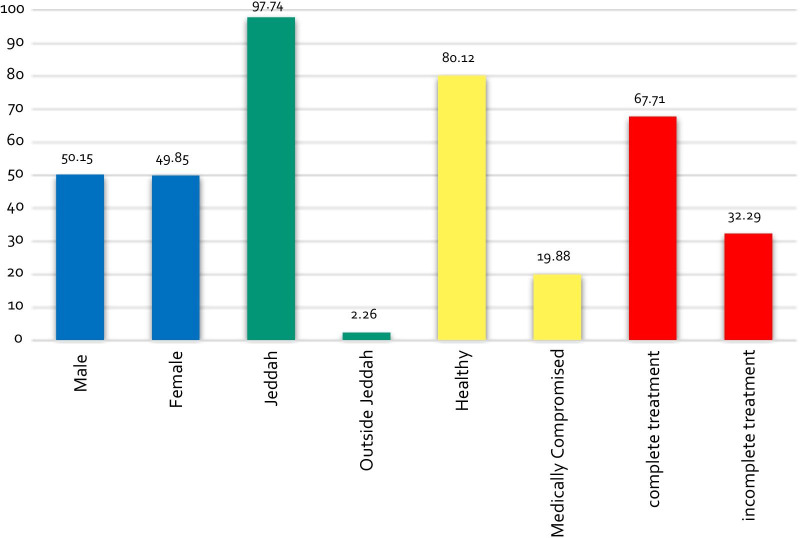
Demographic data of the pediatric dental patients treated at the KAMC-Jeddah

The ages of the dental patients ranged from 0.5 to 17 years, with a mean age of eight years. Majority of the patients were from Jeddah (97.74%) and were healthy (80.12%) at the time of presentation. About two-thirds of them (65%) were treated using the non-pharmacological methods (Npharm), while the remaining one-third (35%), with pharmacological methods (Pharm). The completed treatment summed up to 68% of the total cases (Fig. 1).

**Fig. 2:**
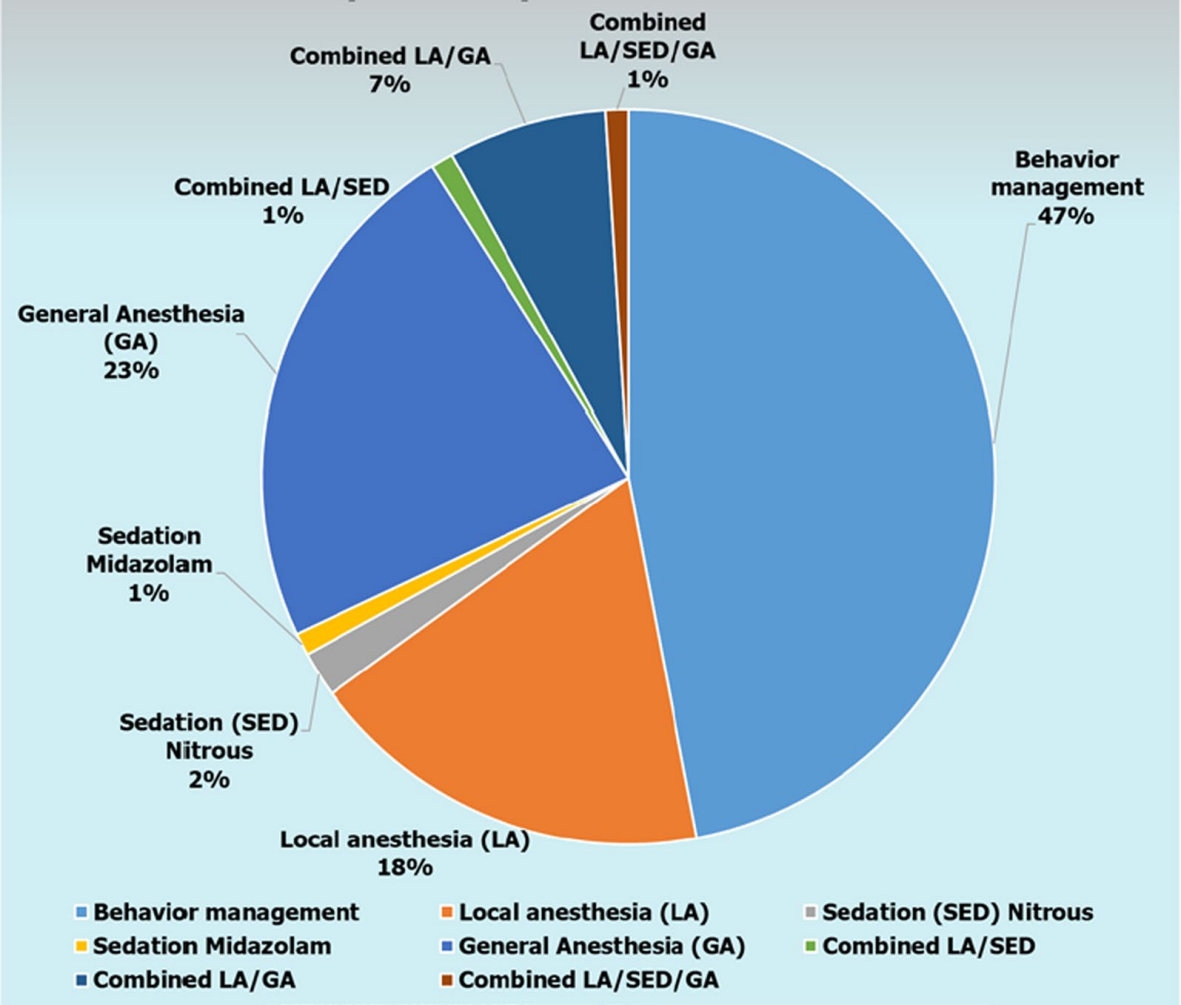
Frequencies of the strategies for the dental treatments used on the pediatric patients at the KAMC-Jeddah

At the KAMC, the average booking for a pediatric patient to complete a dental treatment ranged from 1 to 12 visits, with a mean of 2.68 (± 2.36). The maximum number of dental services provided for each patient was 36 services, and the mean was at 5.74 (± 6.92). Figure 2 lists the frequencies of the strategies used on the dental treatments of the patients. Almost half of the cases reviewed (47%) were treatments that used behavior management techniques only, followed by treatments with local anesthesia (LA), which involved 18% of the cases. Among the pharmacological interventions, the application of GA was the most frequently used method, with 23% of the total cases. The sedation techniques with both nitrous oxide and midazolam was used in approximately 3% of the cases. The combination treatment using both pharmacological and non-pharmacological interventions was applied to less than 10% of the total cases.

**Fig. 3:**
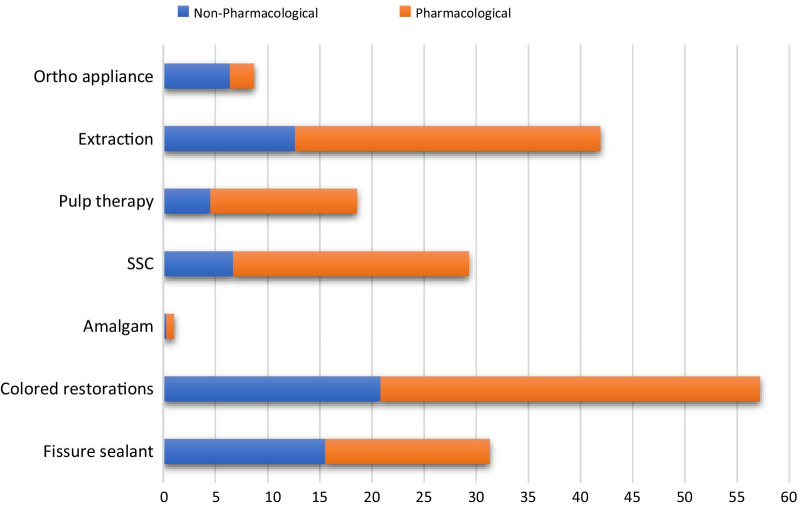
Type of dental treatment under pharmacological and non-pharmacological strategies. (*SSC* stainless steel crowns)

The frequencies of the type of dental treatment provided was significantly associated with the kind of strategy used. The Kruskal–Wallis test and Wilcoxon two-sample test showed that all types of restorative procedures were carried out in significantly higher frequencies with pharmacological treatment strategy. Also, pulp therapy, extraction, and fissure sealant were engaged also in significantly higher frequencies with the attendant pharmacological strategy. Whereas the treatments with space maintainers and orthodontic appliances were carried out in significantly higher frequencies with a non-pharmacological strategy (Fig. 3).

In addition to the types of dental treatment, other factors that were associated with the choice of treatment preferred by the pediatric dentists are described in Table 1. The pediatric patients in the non-pharmacological group were significantly older. The pharmacological group, however, registered a higher number of completed treatments at 92% of the cases (*p* < 0.0001). Only 55% of the cases had completed the treatment with the non-pharmacological intervention.

**Table 1:** Factors that affect the strategies used in pediatric dental treatments

Variable	Dental treatment strategies		*p*-value
	NPharm	Pharm	
	N (%)	N (%)	
*Demographic data*			
Age	8.14 (2.74)	6.83 (2.27)	< 0.0001^a^
Gender			
Male	559 (50.13%)	305 (50.16%)	0.99
Female	556 (49.87%)	303 (49.84%)	
Medical condition			
Healthy	913 (81.74%)	469 (77.14%)	0.02^a^
Medically compromised	204 (18.26%)	139 (22.86%)	
Area of residence			
Jeddah	1,093 (93.03%)	591 (97.20%)	0.27
Outside Jeddah	22 (1.97%)	17 (2.80%	
*Pediatric dental service*			
Treatment completion			
Completed	611 (54.70%)	557 (91.61%)	< 0.0001^a^
Not completed	506 (45.30%)	51 (8.39%)	
Total treatment visit	1.98 (1.42)	3.06 (1.57)	< 0.0001^a^
Total dental treatment services	2.09 (3.87)	12.44 (6.26)	< 0.0001^a^

^a^The significance level was set at 0.05%

The gender factor did not have any influence on the kinds of treatment strategy that were used. The medical conditions of the patients, however, were found to have an influence on the kind of treatment administered (*p* = 0.02). The total number of dental services tendered and the overall number of visits for the treatments were significantly related to the pharmacological strategy that was used (*p* < 0.0001).

The logistic regression statistical model was used to predict the suitability of the treatment strategy provided, i.e., with non-pharmacological or pharmacological strategy, based on one or more independent variables, as shown in Table 2. This model demonstrated that the factors, such as the ages of the patients, the completion of their treatments, the types of treatment applied, i.e., restorative treatment, extraction, or orthodontic treatment, showed significant associations with the treatment strategy. The probability of using the non-pharmacological technique increases with age and the application of orthodontic treatment, posting a positive and direct relationship with each other. On the other hand, the probability of using the non-pharmacological technique decreases with the number of completed treatments and the application of restorative treatment or extraction, giving an inverse relationship.

**Table 2:** Regression model of the significant predictors for pediatric dental treatment interventions

Factors	Odds ratio (CI)	*p*-value
Gender	0.982 (0.692–1.393)	0.9185
Age	1.250 (1.163–1.342)	< 0.0001^a^
Area of residence	1.430 (0.477–4.286)	0.523
Medical condition	1.403 (0.917–2.146)	0.1188
Treatment completion	0.235 (0.153–0.359)	< 0.0001^a^
Preventive treatment	1.107 (0.594–2.060)	0.7495
Restorative treatment	0.381(0.239–0.608)	< 0.0001^a^
Extraction	0.157 (0.102–0.242)	< 0.0001^a^
Orthodontics treatment	1.938 (1.008–3.727)	0.0474^a^

^a^The significance level was set at 0.05%

## Discussion

This study was carried out in a tertiary medical center that provides free dental care to patients, who are eligible based on defined criteria. The center also receives a large number of referrals from in-patient units, out-patient clinics, and primary healthcare centres, scattered all over the western province of Saudi Arabia. The reasons for these referrals point to the specific needs of young patients, who require extensive treatments and/or special attention and preparation due to their behavioral problems or their specific medical conditions. There is an increase in the demand for dental management using pharmacological methods.

Accordingly, this study determined the number of pediatric dental patients and the frequencies of their treatments using pharmacological and non-pharmacological interventions. The comparison between these strategies in managing DFA in children and adolescents has not been sufficiently addressed and studied.

In our study, only 35% of the patients were treated via the pharmacological strategy (Fig. 2). In previous studies, this low percentage was attributed to the following reasons. For example, the parental preference towards non-pharmacological methods was demonstrated by a qualitative study on the attitudes of Chinese parents toward the oral health treatment of their children with caries. This preference was driven by fear of the adverse effects of pharmacological methods and by the lack of dental education of the parents [18]. The medical condition of a patient may contraindicate the use of a pharmacological method. If this is the case, then this can lead to the consequent cancellation of the scheduled dental procedure [19]. Another important reason relates to the limited available slots in the operating room (OR) and the difficulty in scheduling patients for elective dental surgeries in comparison to other critical surgeries from other specialties, which leads to a prolonged waiting time for a dental appointment. This concern was presented and addressed in literature by using a prioritization system that improved the timeliness of treatment for urgent cases, in addition to other measures that can reduce waiting time [20].

Despite the low percentage of the cases in this study that were dealt with using the pharmacological strategies, the completion of their treatments was very high at more than 90% (Table 1). On the other hand, 65% of the cases were addressed using the non-pharmacological strategies, but the completed treatments here were only 55%. A study in Norway explored the explanatory factors that were related to the avoidance or the non-completion of treatments among adolescents, aged 12–18-year old. The canceled or missed appointments led to incomplete treatments, and eventually, caused the future dropping out of the patient from dental care. Here, the canceled or missed appointments constituted as major factors, comprising 47% and 26% of the failures in dental appointments, respectively [21]. Other reported contributing factors leading to incomplete dental treatments include forgotten dental appointments, the negative belief of the dentists, high incidence of caries, painful or unpleasant treatment experiences, and DFA [22, 23]. In our study, it could not be ascertained why the treatments remained incomplete. It was assumed, however, that canceled or missed appointments could be one of the reasons behind this. The very low financial cost and the ease of accessibility to dental care could be excluded as reasons for canceled or missed appointments, because the center provides free dental care services.

There is a growing amount of evidence that a previous painful experience from a treatment is one of the significant factors that triggers DFA in children, which may result in the losses of treatment time or failures in performing the dental procedures [24]. A Swedish study assessed the attitudes of dentists towards pain and their DFA management strategies in children and adolescents [25], and found out that majority of them preferred non-pharmacological strategies. Their results are similar to the results of our study, where the majority of the patients (65% of the cases) were treated using non-pharmacological strategies. Local anesthesia was used only in 18% of the cases (Fig. 2). It is worthwhile to emphasize that LA is an important technique used for pain control in dental treatment in children. It can be administered using alternative delivery systems that aim to reduce fear and anxiety [26]. However, it was found that ineffective pain control is usually associated with dental anxiety, presence of symptoms before treatment, and invasive operative and endodontic procedures [27]. Therefore, the use of LA is sometimes combined with pharmacological interventions to improve the outcome of the dental treatment. Combined application of LA and inhalation sedation resulted in lower levels of post-operative dental anxiety experienced by children having dental extractions [28]. Similarly, the combined use of LA during dental rehabilitation under GA has some potential benefits such as improved patient recovery by decreasing the postoperative pain and hemorrhage control [29]. However, LA use in our study (18%) was categorized under non-pharmacological intervention. This was explained by the characteristics of pediatric patients in non-pharmacological intervention group. Those patients were older in age with more simple restorative procedures and space maintainers. On the other hand, less invasive procedures such as, fissure sealant, and application of topical fluoride in periodic recall visits required only behavioral management techniques as tell-show-do technique and positive reinforcement which comprised the 47%.

Our study determined the factors associated with the choice of the technique preferred by the pediatric dentists. Restorative treatments and extractions were less used in patients, who were treated with accompanying non-pharmacological interventions. Other studies confirmed our findings that children with behavior management problem (BMP) had more carious and fewer filled teeth [24].

As shown in Table 1, the frequencies in using pharmacological techniques in our study decreased with age, i.e., the younger the age of the patient, the more likely a dentist uses sedation or GA, because younger children tend to show BMP [30, 31]. Children’s fear of injection is another reason for the infrequent use of local anesthesia during their treatments, which account for only 18% of the total cases in our study (Fig. 2. This finding agrees with those reported in other studies that children with BMP are fearful of injections. Dentists are also cautious in convincing children or their parents to use local anesthesia in their treatments. This commonly leads to the postponement of receiving dental services or to missing appointments [23, 32–34], which was also reflected in the results of our study.

General anesthesia was used in 23% of the cases. The treatments were completed in all of these cases, with a significantly higher number of restorations and extractions that were done, which also indicated the increased degree of severity of the carious lesions in this group. The number of completed treatments in cases under GA was more than those of the other pharmacological interventions combined. This could be due to the fact that a dental treatment under GA is usually completed during a single visit, compared to other methods, which may require several visits for a treatment to be completed, depending on the extent of cooperation of the patient [35].

The choice of treatment intervention that was used in this study was significantly influenced by the systemic condition of the patient, i.e., whether the patient is healthy or medically compromised. The treatment under GA appears to be more appropriate for children with bleeding disorders, cancers, attention deficit hyperactivity disorders, and other systemic disorders. They require specific attention, and they are more prone to develop DFA during their frequent dental visits [36–38].

Approximately 47% of all cases in this study were treated using behavior management techniques only LA. Based on the regression model, these techniques were associated with the increased chances for orthodontic treatment using space maintainers and orthodontic appliances, and lowered possibility for restorative treatments and extractions. This finding supports our previous finding that children treated using behavior management techniques are older in age. Therefore, most of the treatment visits were dedicated to orthodontic space management, and preventive treatment such as fissure sealants and topical fluoride application. Another explanation for this finding is the fact that since this is a cross-sectional study, some of the children treated with non-pharmacological intervention were treated originally under GA earlier. Hence, during the time of data collection, they were of an older age with less need for restorations and more need for space maintainers and preventive procedures.

The primary limitation of this cross-sectional study design is that the relationship between exposure (treatment strategy) and outcome (dental treatment type) is not time-related because the exposure and outcome are assessed simultaneously. Moreover, even if the association between an exposure and an outcome was determined, there is no evidence that the exposure caused the outcome. In addition, retrospective studies cannot assess all factors that influence the choice of treatment by the dentist. A second general challenge for our study is the external validity since the study results can’t be generalized to a more universal population [39].

Future research should explore further the causes of incomplete treatments among children using the non-pharmacological interventions and the success rates of dental treatments with pharmacological interventions. Also, history of previous dental rehabilitation under GA can be added to data collection for better interpretation of the results and identification of additional factors the influence the choice of intervention.

## Conclusions

Our study documented that pediatric dentists in the KAMC-Jeddah have been commonly treating children using non-pharmacological methods, but in contrast, significantly more dental procedures were administered, and more completed cases were attained by using the pharmacological methods. Most of the allocated clinical time was consumed for preventive care and simple treatment services.

## Data Availability

The datasets used and/or analyzed during the current study are available from the corresponding author on reasonable request.

## Abbreviations

AAPD: American Academy of Pediatric Dentistry
BMP: Behavior management problem
DFA: Dental fear and anxiety
GA: General anesthesia
KAMC: King Abdulaziz Medical City
LA: Local anesthesia
NPharm: Non-pharmacological
OR: Operating room
Pharm: Pharmacological
SED: Sedation technique
SSC: Stainless steel crowns
